# Of city and village mice: behavioural adjustments of striped field mice to urban environments

**DOI:** 10.1038/s41598-020-69998-6

**Published:** 2020-08-03

**Authors:** Melanie Dammhahn, Valeria Mazza, Annika Schirmer, Claudia Göttsche, Jana A. Eccard

**Affiliations:** Department of Animal Ecology, Institute for Biochemistry and Biology, University of Potsdam, Potsdam, Germany

**Keywords:** Behavioural ecology, Urban ecology

## Abstract

A fundamental question of current ecological research concerns the drives and limits of species responses to human-induced rapid environmental change (HIREC). Behavioural responses to HIREC are a key component because behaviour links individual responses to population and community changes. Ongoing fast urbanization provides an ideal setting to test the functional role of behaviour for responses to HIREC. Consistent behavioural differences between conspecifics (animal personality) may be important determinants or constraints of animals’ adaptation to urban habitats. We tested whether urban and rural populations of small mammals differ in mean trait expression, flexibility and repeatability of behaviours associated to risk-taking and exploratory tendencies. Using a standardized behavioural test in the field, we quantified spatial exploration and boldness of striped field mice (*Apodemus agrarius*, n = 96) from nine sub-populations, presenting different levels of urbanisation and anthropogenic disturbance. The level of urbanisation positively correlated with boldness, spatial exploration and behavioural flexibility, with urban dwellers being bolder, more explorative and more flexible in some traits than rural conspecifics. Thus, individuals seem to distribute in a non-random way in response to human disturbance based on their behavioural characteristics. Animal personality might therefore play a key role in successful coping with the challenges of HIREC.

## Introduction

Urbanisation is one of the fastest-occurring and most widespread human-induced environmental changes (e.g.^1,2^). As urban environments expand more and more across the globe, wildlife must either adjust to rapid changes and human-modified landscapes, or experience severe declines and, ultimately, local extinction (e.g.^2,3^). The urban environment presents wildlife with novel challenges, including an altered biotic environment with anthropogenic disturbances, modified competitive interactions, new predators and parasites, as well as altered abiotic factors such as water, soil, light and air pollution, noise, soil sealing, fragmentation and traffic (e.g.^2,4,5^). As a result of these human-induced rapid environmental changes (HIREC^5^), ecosystems are experiencing sharp declines in biodiversity worldwide (e.g.^1,2^). A few species, however, are thriving and occur in high numbers in urban environments. A fundamental focus of current ecological and evolutionary research is to illuminate the drivers of the success of some animals in an urbanised world^3,6,7^ because these “urban laboratories” might advance our understanding of fundamental eco-evolutionary processes and key theoretical concepts, including niche construction and community assembly as well as the role humans play in eco-evolutionary dynamics^8^. The ability of an animal to adjust to novel challenges is likely to contribute to its ultimate success in urban environments (e.g.^3–5,9^). Behavioural adaptations are expected to play a major role in coping with HIREC because behaviour largely determines how individuals interact with their surroundings (e.g.^4,6,9,10^) and behaviour links across fundamental levels of organisation from individual responses to population and community changes^7^. Also, behavioural responses typically occur faster, and are more rapidly reversible, than other responses to environmental change (e.g.^5,7^). In fact, behaviours of the so-called “urban adapters” often differ from those of their same-species rural counterparts (e.g.^3,5,9,10^). Two main factors are usually considered as potential drivers of differences in behavioural responses to human disturbance in urban wildlife: behavioural flexibility and intrinsic behavioural characteristics (e.g.^3,5,9^). Behavioural flexibility (or reversible phenotypic plasticity) might allow some animals to habituate faster than others, become less sensitive to novel threats and find new alternative solutions to the challenges of urban life (e.g.^3,5,9^). Several studies have demonstrated elevated behavioural flexibility within urban-adapter species (e.g.^11–14^). However, research also suggests that some differences in the behaviours of urban adapters cannot be explained by habituation/plasticity alone (e.g.^15,16^). The second potential driver of behavioural adaptations to urban environments are intrinsic behavioural characteristics (e.g.^3,5,9^). Some individuals might just be better suited to reach and successfully colonize urban habitats than others (e.g.^3,5,9^). Between-individual behavioural differences that are stable over time and across contexts are termed animal personality^17^, temperament^18^ or coping style^19^. Individual animals can exhibit, for example, consistent levels of exploration, boldness, activity, sociability and aggression (e.g.^17^). Individuals with different behavioural types can play different ecological roles (e.g. exploiting different resources or ecological niches, being favoured at different stages of the dispersal/colonisation process) (e.g.^20,21^). “If some personality types are better suited to dealing with certain challenges than others, personality could determine how successfully individuals are able to occupy a range of different environments with different selective pressures”^22^. Following this line of reasoning, individual differences in spatial exploration/dispersal tendencies, risk-taking and tolerance to human disturbance might promote the successful colonisation and settlement in urbanised habitats of only certain behavioural types (e.g.^9^).

Among the axes of behavioural variation presenting potentially important implications for dispersal, settlement and establishment in novel environments, and particularly for coping with human-altered environments, are boldness and spatial exploration. Boldness is a personality trait that is characterized by individual differences in willingness to take risks in a variety of contexts (e.g.^23–25^). Here, we define risk-taking as the behaviour expressed in a risky situation and boldness as consistent individual differences in risk-taking behaviour^17,26^. Since behaviour under risk is a key determinant of both components of fitness (e.g.^26,27^), it is likely to be an ecologically relevant personality trait. It is well established that boldness has fitness consequences (e.g.^28^), is heritable (e.g.^29–31^), and subject to selection (e.g.^32,33^). Furthermore, boldness is linked to life-history decisions such as dispersal (e.g.^34,35^), foraging (e.g.^26,36,37^), antipredator behaviour (e.g.^24,36,38^), and mating (e.g.^39–41^).

Exploration refers to the gathering of information about the environment^42^, although the same term is also used to indicate the reaction to unfamiliar objects and places^17^. When applied to spatial exploration, a common problem faced by behavioural ecologists is to distinguish between the general locomotor activity and the gathering of environmental information (e.g.^43–45^). While the distinction is particularly sensitive for species that might use vision as a primary source of information, for taxa that rely predominantly on olfaction and touch to explore their environment/space, inter-individual variation in activity and spatial exploration are generally considered functionally integrated (e.g.^43,46^). Here, we define exploration as the gathering of information about the environment (e.g.^42^) and refer to spatial exploration as consistent individual differences in exploratory behaviour in the context of space and in connection to movement (e.g.^43,46^), and use the term “general activity” for locomotion. Exploration presents a heritable component (e.g.^47–51^), affects survival (e.g.^28^) and reproductive success (e.g.^52–54^), indicating that inter-individual variation in exploration could create targets of selection^43^, and that it is likely to be an ecologically relevant personality trait. Furthermore, inter-individual variation in spatial exploration predicts dispersal tendency and space use in a diverse range of taxa (e.g.^9,55–61^), shares a genetic underpinning with dispersal, at least in some species (e.g.^50^), and is also suggested to facilitate the range expansion and invasive spread into new habitats (e.g.^43,62^).

Investigating the differences in boldness and spatial exploration between rural and urban populations might therefore help to illuminate the determinants of successful adaptation to urban colonisation, as well as reasons why most animals fail to adapt to rapidly changing environmental conditions. In the past few years, studies investigating behavioural adaptations to urban environments have flourished, focusing particularly on avian species (e.g.^22,63–65^).

Here we focus on small, ground-dwelling species with more restricted dispersal abilities and aimed at testing whether urban and rural populations of a widely distributed rodent differ in behavioural flexibility and in intrinsic behavioural characteristics. Understanding the determinants of successful urbanisation of rodents is of particular interest because many species are urban dwellers or even synanthropic and reservoirs for a variety of human pathogens (e.g. hanta virus, e.g.^66^). Our study species was the striped field mouse (*Apodemus agrarius*), an omnivorous small rodent common in central and Eastern Europe and Asia. Striped field mice are known to successfully colonise urban environments (e.g.^67^). Differences between rural and urban striped field mice have been reported regarding diet composition (e.g.^68^), morphological traits (e.g.^69,70^), demography and population dynamics (e.g.^71^), genetic structure and genetic differentiation (e.g.^72^), suggesting that striped field mice do occur in both environments and adjust to urban habitats. Striped field mice thus make a suitable study species for investigating potential intra-specific behavioural differences between rural and urban dwellers. Specifically, we hypothesised that (1) rural and urban populations differ in mean trait expression of spatial exploration and boldness, i.e. in animal personality, and (2) in flexibility in these two traits.

We predicted (i) that striped field mice would show consistent between-individual differences in boldness and spatial exploration. Furthermore, we predicted (ii) that urban individuals would show higher levels of boldness and spatial exploration compared to their rural conspecifics and that (iii) trait expression increases with increased sealed surface, our proxy for urbanisation. Lastly, we expected (iv) behavioural flexibility to be more pronounced in urban animals compared to rural conspecifics.

## Results

**Figure 1:**
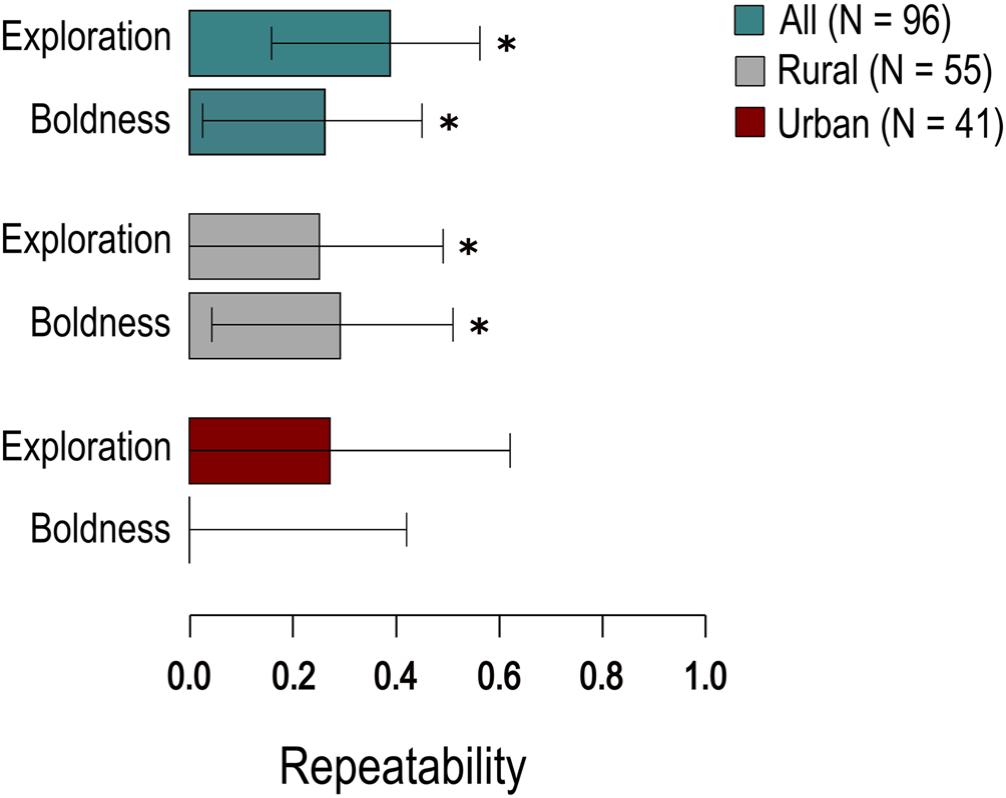
Repeatabilities of composite behavioural variables for striped field mice (*Apodemus agrarius*) quantified in short behavioural tests on-site. Asterisks represent significant deviation from zero.

We captured 96 striped field mice, 55 of which in the rural sites and 41 in the urban sites; of these, 56 individuals were trapped and tested multiple times (average ± SD number of tests per individual: 1.75 ± 0.74). At dataset level, both spatial exploration (*R* = 0.39, 95% CI = 0.16–0.56) and boldness (*R* = 0.26, 95% CI = 0.02–0.45) were repeatable over time (Fig. 1 and Table S4 in the supplementary material). While habitat-specific repeatabilities were based on small sample sizes, they suggested that both behavioural traits might be more repeatable in rural than urban habitats (see Fig. 1 showing indices and Fig. S2 and Table S4 in the Supplementary Material showing original estimates).

**Figure 2:**
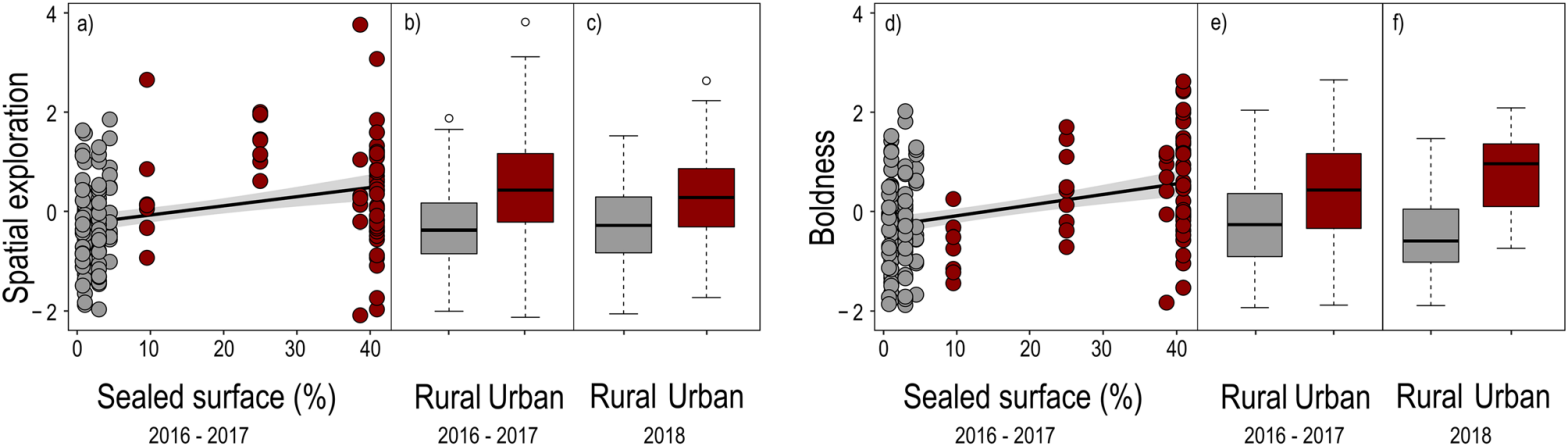
Spatial exploration (**a**) and boldness (**d**) of 96 striped field mice (*Apodemus agrarius*) increase with increasing percentage of sealed surface in a 1 km buffer (i.e., urbanisation). Predictions (line) and confidence bands (grey shading) are based on LMMs including all animals. The boxplots show spatial exploration (**b**) and boldness (**e**) for the pooled populations of rural (N = 55) and urban (N = 41) striped field mice analysed in the present study. The boxplots (**c**) and (**f**) show similar patterns for spatial exploration and boldness for pooled populations of rural and urban striped field mice tested within the same study year. These data are not included in the main analyses.

Urban striped field mice were characterised by higher spatial exploration (*β* = 0.73 ± 0.18, *t* = 4.07, *P* < 0.001—Fig. 2 and Table S1 in the Supplementary Material) and boldness (*β* = 0.55 ± 0.18, *t* = 3.10, *P* = 0.003—Fig. 2 and Table S1 in the Supplementary Material) than rural conspecifics. Spatial exploration and boldness also increased with increasing sealed surface, our proxy for urbanisation (spatial exploration: *β* = 0.02 ± 0.01, *t* = 3.08, *P* = 0.003; boldness: *β* = 0.02 ± 0.005, *t* = 4.15, *P* < 0.001—Fig. 2), although when restricting the analysis to the urban subset only boldness increased with increasing sealed surface (*β* = 0.04 ± 0.012, *t* = 2.94, *P* < 0.003—Table S6 in the Supplementary Material). Results for the single behavioural variables are reported in the Supplementary Material (Tables S1 and S2).

Individual intercepts and slopes differed between rural and urban animals for both spatial exploration (intercept: W = 154, *P* < 0.001; slope: W = 490, *P* = 0.026) and boldness (intercept: W = 208, *P* = 0.009; slope: W = 482, *P* = 0.037), with urban individuals having steeper slopes compared to rural conspecifics (Table S5 in the Supplementary Material). Results for the single behavioural variables are reported in the Supplementary Material (Table S5).

Given the potential confounding effect of sampling year and rural/urban habitat, we conducted an additional analysis on a separate dataset that was collected at a subset of the original urban and rural sites within the same year (i.e., without a year confound). This corroborative analysis showed a consistent pattern in behavioural responses and PC components spatial exploration and boldness (Fig. 2, Table S7). Spatial exploration was higher in urban animals (*β* = 0.67 ± 0.22, *t* = 3.09, *P* = 0.003—Tables S11 and S12 in the Supplementary Material) also when considering the first round of testing only, when the arena was entirely novel to all animals.

## Discussion

Based on repeated behavioural assessment, we found that boldness, spatial exploration, and behavioural flexibility in striped field mice differ between rural and urban habitats. Overall, striped field mice exhibited significant repeatability in most behavioural responses measured, indicating individual consistency over time in traits related to boldness and spatial exploration. According to our predictions, behavioural responses connected to spatial exploration and boldness significantly increased with level of urbanisation. Also, urban animals showed higher levels of reversible behavioural plasticity.

As predicted, urban striped field mice were more explorative than conspecifics from rural sites. Spatial exploration is likely to be a key trait for all stages involved in the colonisation of a new environment (e.g.^9^). As urbanization increases, it creates fragmented landscapes that only highly active and explorative animals are able to reach (e.g.^73,74^). Thus, there should be an environmental filter favouring explorative individuals to arrive in the urbanised areas in the first place. Explorative individuals commonly use larger home ranges (e.g.^75^) and disperse farther from their natal area (e.g.^35,55,62^). They are also more likely to discover and exploit important resources (e.g.^76^). Highly explorative individuals would then be favoured not only in the arrival stage of colonization, but also during establishment and increase due to the continued exposure to novelty (e.g., food type and availability, space and habitat alterations, heterogeneity in disturbance^3,9^). The composition of urban populations could thus stem from a subset of more explorative and active individuals colonizing urban fragmented areas (e.g.^64,74^). Spatial exploration scaled positively with the degree of urbanization only when considering the complete dataset, but this effect was no longer present when analysing the urban data only. However, most of the single behavioural responses loading on the composite variable spatial exploration scaled positively with sealed surface. It is therefore unclear whether this is an effect of reduced statistical power, and/or the different weight that the single variables carried to the composite score of spatial exploration. Alternatively, animals with more pronounced explorative tendencies might be more likely to disperse and colonise cities but then the degree of spatial exploration might no longer be favoured within urbanised environments.

Predator numbers tend to increase with urbanization, whereas the predation pressure on urban prey populations is often reported to be lower than in nonurban areas—a phenomenon known as the predation paradox^77,78^. Urban areas are in fact being colonised not only by small rodents, but also by many of their predators, such as foxes, coyotes, stoats and birds of prey, that even achieve higher population densities than are found under natural conditions (e.g.^79,80^). In the present study, urban striped field mice were bolder than rural conspecifics and boldness scaled positively with the degree of urbanization. Reported results of increasing boldness with increasing urbanisation are consistent with the results of previous studies, which suggested that a higher risk-taking tendency is adaptive during invasion of novel environments (e.g.^65,81^, but see^82^). Higher boldness, defined as an individual’s reaction to any risky situation, including encounters with predators and humans^17^ is thus likely to help coping with the threats and challenges posed by “city life”, from traffic and other human disturbances to responses to higher numbers of predators. Indeed, increased boldness along the rural–urban gradient may arise as a consequence of selective pressures precluding shy, fearful individuals from colonizing urban areas (e.g.^63,64,83^). Bold individuals are also more likely to arrive in new areas because they disperse further and pass through the environmental filter of potentially hostile environments. It is not clear, though, whether high boldness would be always equally adaptive in the establishment and increase stages of the colonisation (e.g.^84^). The dangerous niche hypothesis^85^ proposes that heightened caution is adaptive when novel stimuli are likely dangerous, e.g. if toxic foods, traps or a high level of predation risk characterize an individual’s environment^84,85^. Thus, while reduced risk-taking tendencies may be adaptive during the colonization of cities (e.g.^86,87^), persisting there might necessitate more cautious behaviour^84^. Differential selective pressure in different stages of colonisation might explain why in some cases urban individuals show similar risk-taking to their rural counterparts (e.g.^82,84,88^). Long-term and population genetics data alongside behavioural assessments are needed to assess if and how such a shift in favoured conditions takes place.

Behavioural flexibility implies a rapid response to novel environmental conditions and presumably allows animals to exploit a wider variety of ecological contexts^89^. Urban striped field mice showed higher levels of behavioural flexibility in spatial exploration and boldness. Exploration has a positive and direct effect on habituation (e.g.^16^), one of the most common forms of behavioural plasticity (e.g.^90^). Habituation to novel stimuli is also considered a simple form of learning (e.g.^90^), which is also often linked to exploratory tendencies and boldness (e.g.^91,92^). Exploratory behaviour is an important means to acquire information about environmental properties and make later decisions (e.g.^93,94^). Being able to habituate faster would favour the colonisation and the establishment of new human-altered environments. In the same way, making rapid assessments of what constitutes a threat in the novel environment and what can instead be safely ignored would prove advantageous, effectively lowering stress levels and allowing optimal resource allocation (e.g., of attention or vigilance). Thus, colonization of urbanised habitats could be facilitated if the dispersal ability of explorative individuals is coupled with a greater potential to habituate (e.g.^16,55^). This being the case, being bold and having a higher tolerance to disturbance would certainly favour urban success, but being too bold might prove maladaptive and considerably shorten an individual’s life-expectancy (e.g.^28^). Similarly, consistent high levels of spatial exploration carry costs, for example in increased chance of picking up parasites (e.g.^95,96^), running into predators or even not reacting fast enough to predator attacks (e.g.^75,97^). Also, discovering and exploiting novel resources, considered one of the main advantages of being explorative and bold, might lead to poisonous/poisoned food (e.g.^98^). Therefore, being able to flexibly adjust the behavioural response would indeed prove beneficial (e.g.^99^), especially in the case of a small mammal subject to intense predatory pressure, and potentially exposed to poisons intended for other rodents’ management and control (e.g.^100^). The ability to adjust might explain why in our study the repeatabilities of boldness-related behaviours on urban sites were low. Further, sample size was likely affecting the repeatability at habitat-level (rural/urban). Similarly, previous studies reported more variation in the expression of measured behavioural responses in urban individuals compared to rural ones (e.g.^22,84,101^). For example, Lehrer et al.^101^ found increased variance of vigilance behaviour in woodchucks (*Marmota monax*) along the rural–urban gradient. They suggested that urbanization increases spatial heterogeneity in perceived predation risk for wildlife species^101^. When the environment is subject to a relatively high rate of change and variability, enhanced behavioural flexibility is a key factor in determining an individual’s fitness (e.g.^9,102^).

We acknowledge that comparing rural and urban data from consecutive years might result in a potential confound between study year and study areas, if, for example, weather conditions which impact on food availability varied between years, and these in turn would favour different behavioural types (e.g.^29,103^). However, such alterations in abiotic conditions can also occur at the same time at a smaller local scale (e.g.^104,105^). Additionally, cities present specific microclimatic conditions that make them more similar to other cities than to the natural landscape surrounding them (e.g.^73,106^). Urban features such as reduced land and vegetation cover and energy use, along with the extent and configuration of structures such as buildings, streets, and parks shape weather patterns and air quality, altering atmospheric flows, humidity, precipitation, and temperatures (e.g.^106,107^), which are usually warmer than in rural areas (e.g. ^106,108,109^). Such ecological differences are an intrinsic part of all comparisons between rural and urban environments. We are confident that the observed differences between rural and urban populations are robust patterns because an additional analysis carried out on a separate dataset from a study conducted in the same rural and urban sites within the same year indicates that the same patterns are maintained when data are collected during the same season and under similar conditions (or as similar as they can be, given the caveats described above).

At present, we are unable to determine which mechanisms underlie the observed differences in spatial exploration and boldness between rural and urban striped field mice. Sol et al.^9^ suggested three potential mechanisms that would explain the widespread patterns of behavioural adjustments to urbanisation: (1) higher behavioural plasticity in urban individuals compared to rural ones, (2) non-random sorting (only some behavioural types could successfully colonize novel environments), and (3) evolutionary change via divergent selection. Whilst there is a substantial body of research reporting behavioural modifications in urban wildlife, it is often unknown whether the observed behaviours are inherently plastic or the product of genetic adaptations^3^. A genetic heritable base is required for any focal trait that undergoes selective pressures; numerous studies have shown that consistent behavioural traits do have a heritable component, and that natural selection can act on them (e.g.^47,52^). However, studies assessing such pressures or fitness-related aspects of different behavioural types in the context of urbanisation are still rare (but see e.g.^110^).

Further data on the degree of genetic isolation of urban populations and the direction of gene flow between rural and urban areas are also needed to assess whether constant influx of preferentially bolder behavioural types into urban populations occurs^110^. It has been suggested that different behavioural types may be favoured at different stages of the urbanisation process (e.g.^9,110^). Interpretation of findings and insights into apparently mixed results will benefit from a precise understanding of how advanced the colonization process is for the target species. Historical trapping data along with genetic analyses to determine genetic distance across neighbouring populations can provide valuable insights. Future studies adopting a common garden approach will shed light on whether animals originating from areas with different degrees of urbanisation yet born and raised in the same conditions display genetically-based differences in personality traits (e.g.^65^). Conversely, if the behavioural differences that arise along the urban gradient are the consequence of behavioural plasticity, it would be interesting to investigate whether behavioural plasticity itself is heritable and selected for in the urban landscape (e.g.^9^). Lastly, it will be interesting to investigate how these behavioural traits link to other, slower-evolving aspects, like physiological and morphological adaptations (e.g.^83,110,111^).

In conclusion, this study suggests that behavioural adjustments can play a functional role in coping with urbanisation, one of the most widespread human-induced environmental changes. Our results are congruent with the interpretation that urbanization might affect the distribution of individually consistent behaviours, favouring individuals with specific personality traits and behavioural flexibility. To our knowledge, this is one of the first empirical studies testing a general hypothesis on individuals showing behavioural differences in connection to urbanisation in a small, non-commensal mammal species. Findings are consistent with previous literature on birds and larger mammals, suggesting an ubiquitous response to human-altered environments across taxa. Urbanisation may therefore act as a selective force driving the evolution of behavioural and phenotypic differences between urban and rural populations^22^. Cities provide a natural laboratory for understanding which role human activity plays in the reciprocal interactions between ecological and evolutionary processes, since the accelerated rates and increased magnitude of landscape-level changes mark them as hotspots of contemporary evolution^110,112^. Urban environments proved to be suitable settings, not only to study how personality traits might influence the success in human-altered landscapes, but also to give insights into the complex relationship between behavioural consistency and plasticity. Additionally, the study of animal personality could help understanding how animals cope with human-induced rapid environmental change.

## Methods

### Study sites

The study was conducted in four different urban sites in Berlin (52° 31′ N, 13° 24′ E, area 891.1 km^2^), with varying degrees of anthropogenic influences, and five rural sites located in a region of NE-Germany called Uckermark (53° 35′ N, 13° 71′ E, area 3,058.2 km^2^). All sites were part of the *CityScapeLabs* and *AgroScapeLabs* experimental platforms (https://www.bbib.org). With over 3.5 million inhabitants and an area of 892 km^2^, Berlin is the largest and most populous district of Germany (https://www.statistik-portal.de/Statistik-Portal/gemeindeverz.asp?G=Berlin). Numerous green spaces are separated by dense residential and commercial developments. The Uckermark region is an intensively farmed area, with comparatively low human population densities (39/km^2^) (https://www.citypopulation.de). Urban sites varied in size between 0.75 and 1.16 ha and were characterized by heterogeneous vegetation including grassy areas, bushes and small trees. The rural trapping sites were fallow lands between arable fields, which varied in size between 0.85 and 1.66 ha and were characterized by heterogeneous vegetation composed of grassy areas streaked with nettles, bushes and trees. Rural and urban trapping sites were on average 102.6 ± 3.7 km apart. Urban sites were on average 13.2 ± 6.6 km apart from each other, while rural sites were on average 9.5 ± 5.8 km apart. Striped field mice have an average home range size of 2,737 ± 2,046 m^2^, an average core area of 600 ± 446 m^2^, and an average daily distance travelled of 696.6 ± 311.8 m^60,61^. During our trapping, it never happened that individuals trapped in one site were found in another. We therefore consider a transfer from one site to the other over the course of the study to be extremely unlikely. We used a sealing index, i.e. the coverage of natural soil with artificial impervious surface (e.g. buildings and paved roads), as a proxy of the degree of urbanisation in each site. Sealed surface closely corresponds to other urbanisation indices, such as human population density, disturbance by humans and pets, noise and light pollution (e.g.^113,114^). The index was calculated for a buffer around each study site with radius of 1 km. Sealed surface of Berlin sites was calculated within the *CityscapeLabs* project on the basis of the biotope mapping of Berlin^113,115^. Sealed surface of rural sites was calculated with QGIS^116^.

### Capture-mark-recapture

At each site, we used a capture-mark-recapture approach with 44–56 multiple-capture live-traps (Ugglan Special Traps n. 1–2, Grahnab AB, Hillerstorp, Sweden) set in a regular grid of 10-m distance between traps. Traps were pre-baited with oat flakes and apples for two nights. Once activated, they were checked every morning and afternoon. At the urban sites, trapping was conducted in two sessions with 7–9 days between sessions. Each trapping session lasted approximately 72 h with 4–5 trappings in the first session and 2–3 in the second session. At the rural sites, trapping was performed continuously for several weeks and tests were repeated upon recapture of the individual. The average inter-trial interval was 11.3 ± 13.2 days between first and second test and 10.8 ± 6.5 between successive tests. Upon first capture, individuals were sexed, weighed, marked individually with a unique fur-marking and a set of standard morphometrical measures was taken. First behavioural tests were performed either at first capture (without any prior handling) or at first recapture. In total, 96 individual striped field mice were tested in this study. Of these, 40 could only be tested once and 56 were tested multiple times (of these, 41 individuals were tested twice, 14 individuals were tested 3 times and 1 individual was tested 4 times) for a total of 168 tests. Data collection took place from August to October 2016 (rural sites) and from July to September 2017 (urban sites).

### Behavioural tests

For behavioural testing, we modified standard laboratory tests that are commonly used in personality studies of small mammals (e.g.^18,117^) and combined them^60^ to be executable directly on site, in one run and without prior handling. The set-up combined and adjusted the set-ups of the dark light test^118^ and the open field test^119^. These combined set-ups are used to assess boldness and spatial exploration in a novel environment (e.g.^46^). These tests have been developed for rodents (e.g.^119–122^) and yield biologically meaningful measures of what is intended to measure (e.g.^36,60,61,123^). Albeit a test developed for one species/taxon is not necessarily appropriate as a test for another (e.g.^44,45,124^) the open-field test is a classical test for rodents and behaviours measured in this test have been ecologically validated in many species (e.g.^123^), including the taxon of this study^61^.

The dark–light test measures willingness of individuals to leave a dark and enclosed shelter to enter an unknown, bright and potentially risky area. The open field test was developed to specifically target rodents’ aversion to brightly lit and open spaces^125^, and thigmotaxis, i.e. a tendency to remain close to vertical/peripheral objects instead of in open spaces^122^, presumably to reduce the possibility of predation^126^. It quantifies an individual’s exploratory activity and risk-taking propensity by assuming different levels of perceived risk in different arena parts (e.g. ^60,127^). The open space of the test arena is unprotected which, in the natural environment, would leave a rodent open to overhead predator attacks^122^. As small mammals, striped field mice are vulnerable to both terrestrial and avian predators (e.g.^128–130^), and being on the move (i.e. more visible) or exposed in an open and bright clearing, makes them extremely vulnerable to predation. Leaving a dark, enclosed, protected shelter to enter such a space is an indication of an individual’s propensity to take risks. While some critics would prefer to distinguish between the general activity and the act of exploration, for spatial exploration, especially in a primarily-non-visual animals it might not be possible (e.g.^46^). In order to explore a space, rodents have to move through it, as they mainly rely on their olfaction and whisker-mediated touch system to gather environmental information (e.g.^131–136^), and transmit tactile information for object and texture recognition (e.g.^137–139^) as well as spatial information such as localization in space (e.g.^134,140^), as they sweep the environment by intrinsic muscles^132^).

In their natural environment, small mammals that scored high in the open field test were also the ones that had significantly larger exploratory home ranges, moved greater daily distances, occupied larger home ranges and were more active (e.g.^60,61,123^). Also, small mammals’ higher activity levels in the open field correlate with information acquisition and use (e.g.^127^), use of resources and foraging strategies (e.g.^36^), and mating and reproductive decisions (e.g.^141^). The behaviours assessed in the dark–light test and the open-field test are considered adaptive in the natural environment and variance in the responses can provide ecologically-relevant indicators of behavioural traits like risk-taking propensity or boldness and spatial exploration tendency^122^.

The test set-up we used here consists of a dark plastic tube of 32 cm length and 15 cm diameter with swing doors at both ends connected the trap in which the animal was found to the PVC open field arena (130 cm Ø, 30 cm high) (Figure S1 in the Supplementary Material). Once the animal entered the tube we closed the external swing door; after 60 s we opened the internal swing door and measured the subject’s latency to enter into the open field arena with the head (‘latency head’) and the full body without tail (‘latency body’). If animals did not leave the trap within 1 min, they were gently guided into the dark tube (53% of all performed tests). If the animals did not leave the dark tube within five minutes, we set the latency to 300 s (17% of all performed tests) and animals were gently guided out of the tube into the arena. When the animal entered the circular arena, we closed the tube door and the open field test started.

The floor of the open-field arena was virtually divided into 16 areas of equal surface, although peripheral sections were considered safer than the more exposed central ones. We recorded each animal’s behaviour for five minutes. We measured the following parameters: latency to enter the central area with the full body (excluding tail), time spent active (that is, moving around the arena) or inactive, number of crossings into the central area with the whole body (without tail), number of jumps, number of explored sections. We ensured high inter-observer reliability with test trials prior to data collection with individuals not involved in the study. If animals did not enter the central area within five min, the latency to enter the centre was set to the maximum of 300 s (3.6% of all trials). We conducted all tests by direct observation, between 08.00 and 18.00 h, in the shade and only in rain-free periods. At the end of the test, animals were released at the site of capture.

### Statistical analyses

We estimated repeatability of the recorded behavioural variables, a population-specific metric to quantify inter-individual phenotypic differences across time or contexts, using an LMM with individual as a random factor^142^ using the R package *rpt*R^143^. We calculated 95% confidence intervals (CI) of repeatabilities for each variable by parametric bootstrapping (N = 1,000 simulation iterations)^144^. *P* values were calculated based on 1,000 permutations.

In a first step of the analyses, we compared each behavioural variable across rural and urban populations, since the proportion of sealed surface was very similar for all rural sites. We used repeated measures linear mixed effects models (LMM) and generalized linear mixed effects models (GLMMs) fitted by restricted maximum-likelihood. Individual identity was added as a random factor, specified as random intercept, in each model to control for non-independent repeated measures of the same individual. In prior analyses, we included trapping site as an additional random factor. Since this factor did not improve model fit, according to associated likelihood ratio test (*P* < 0.001)^145^, we retained more parsimonious models without trapping site. In a second step, we used the same statistical approach to evaluate the relationship between behavioural variables and sealed surface as a proxy for urbanisation. This allowed us to control for the two different sampling years and to investigate possible gradual changes along the rural–urban gradient. In a third step, we used the same statistical approach to evaluate the relationship between behavioural variables and sealed surface as a proxy for urbanisation within the urban plots using only the data from urban animals. In all models we added two temporal control variables as fixed effects: DayMCI—the experimental day mean-centred for the individual and the study period to control for variable time periods between repeated tests of individuals and DayMCP—the experimental day mean-centred for the study year to control for potential seasonal variation. Both temporal control variables were also added as quadratic terms to allow for non-linear relationships. Sex of the individual was also added as a fixed effect. We included all possible two-way interactions between the explanatory variables. We excluded all non-significant quadratic terms, interactions, and the fixed effect sex, i.e. all control predictors, based on stepwise backward model selection using log-likelihood ratio tests comparing nested models^145^. Latencies were log-transformed, and the proportion of time intervals animal spent active (from here on general activity) was arcsine-square-root transformed. The number of explored sections was turned into a binary variable of all sections visited or not. Visual inspection of residual plots did not reveal any obvious deviations from homoscedasticity or normality. We used the R packages *lme4*^146^ and *nlme*^147^. We report these results in the Supplementary Material (Table S1 and Table S2).

We used principal component analyses (PCA) followed by *oblimin* rotation^148^ to reduce the number of dependent variables. We retained principal components with *Eigenvalues* > 1 (Kaiser–Guttman criterion^149^). The PCA returned two main components that accounted for 69% of the variance (PC1 = 35%, *Eigenvalue* = 1.75; PC2 = 34%, *Eigenvalue* = 1.57, Table S3 in the Supplementary Material). The first component best explained the variance associated with the number of crossings, jumps and the proportion of time spent active in the arena; it was thus named ‘spatial exploration’ because it explained the general exploration activity combined with movement in the arena. The second component was associated with the latencies to leave the dark tube, enter the central part of the arena and the number of crossings; it was thus named ‘boldness’ because it accounted for each individual’s risk-taking propensity. For each component, we followed the same statistical procedure as described above.

Due to the biases involved in point estimates of random slopes and intercepts using restricted maximum likelihood linear mixed effects models^150^, we followed a Bayesian approach to estimate individual differences in behavioural traits and reversible plasticity in behavioural traits over time (R package *MCMCglmm*, Markov-chain Monte Carlo generalized linear mixed models^151^), which are less biased and allow better estimates of uncertainty. We fitted each behaviour as the response and included DayMCI as a control predictor, fitted as fixed effect. We built a random intercept-slope model with DayMCI fitted as a covariance term to estimate within-individual plasticity of behaviour over time. We used slightly informative priors by dividing the total phenotypic variance of the behavioural trait by the number of random effects (n = 2) in the model and set a low degree of belief (nu = 0.002)^151^. We used 1,010,000 iterations, with a burn-in of 5,000 and a thinning interval of 100. These parameter settings resulted in low temporal autocorrelation between estimates of subsequent models, which were assessed by graphical diagnostics. Subsequently, we extracted individual random intercepts (i.e. best linear unbiased predictors, BLUPs) and individual random slopes (i.e. within-individual reversible plasticity over time) and compared these traits between urban and rural animals with a Mann–Whitney-U test. The accepted significance level was ≤ 0.05. All data analyses were conducted with R version 3.2.3 (R Core Team 2015).

### Corroborative additional analyses

#### Year versus site effects

Given the potential confounding effect of sampling year and rural/urban habitat, we used data from a different study to ascertain whether differences in behavioural patterns could be traced to different environmental conditions in different years or could instead be attributed to the habitat characteristics. In this follow-up study we trapped and tested 42 rural and 25 urban striped field mice in 3 rural and 4 urban sites. These sites were selected among the ones in which the data collection for this work was carried out. Trapping and testing protocols reflect those described above, with small discrepancies that preclude adding these data to the main analysis. In this part of the study we only tested each animal once. These data are used only to ascertain that a similar pattern is present when sampling is carried out in the same year, and are not added to the main dataset that is described in the rest of this work. Patterns are shown in Fig. 2 and Table S7.

#### Repeated testing and habituation

Since behavioural tests were repeated a third or fourth time for rural animals, and this could have affected the results and inflated the repeatability estimates, we ran additional analyses with a subset of the data, using only the first two tests for all animals. We compared each behavioural variable across rural and urban populations, and between behavioural variables and sealed surface as a proxy for urbanisation, using the same statistical procedure as described above for the full dataset. Results are reported in the supplementary material (Tables S8, S9 and S10).

#### Spatial exploration

Since some definitions of ‘exploration’ refers to reactions to novel situations (e.g.^17^) and our main analysis includes also repeated measures of the same animals, we compared PC scores and single variables loading on them of rural and urban individuals during the first trial only, to ensure that the measured responses applied to the unfamiliar arena. We used linear models (LMs) and added MCP as fixed factor. Results are reported in the supplementary material (Tables S11 and S12).

#### Ethical note

Animal capture and behavioural tests were conducted under the permission of the “Landesamt für Umwelt, Gesundheit und Verbraucherschutz Brandenburg” (reference number: LUGV_7RO-4610/34 + 5#86908/2011; V3-2347–44-2011 and RO7/SOB-0998A-C), the “Senatsverwaltung für Stadtentwicklung und Umwelt” (reference number: IIIB2/OA/AS/G1394), and the “Landesamt für Gesundheit und Soziales” (reference number: G 0072/16). Experiments were performed in accordance with all applicable international, national, and/or institutional guidelines for the use of animals, including the ASAB/ABS guidelines for the Use of Animals in Research. Captured animals were released at the capture site directly after testing.

## Supplementary information

Supplementary Information.

## Data Availability

Data are available as supplementary material.
