# Supplementary material for: Of city and village mice: behavioural adjustments of striped field mice to urban environments
Source: Sci Rep. 2020 Aug 3;10:13056. doi: 10.1038/s41598-020-69998-6 (PMC7400609; doi:10.1038/s41598-020-69998-6)
Supplement: Supplementary file 1 — Supplementary Information. [file 41598_2020_69998_MOESM1_ESM.pdf]

SUPPLEMENTARY MATERIAL

Of city and village mice: behavioural adjustments of striped-field mice to urban environments

Melanie Dammhahn*[1], Valeria Mazza*[1], Annika Schirmer[1], Claudia Göttsche[1] & Jana A. Eccard[1]

*These authors contributed equally.

[1]Department of Animal Ecology, Institute for Biochemistry and Biology, University of Potsdam, Potsdam, Germany.

Correspondence to: vamazza@uni-potsdam.de, mdammhah@uni-potsdam.de

Fig. S1 - Schematic representation of the experimental apparatus. All sections of the Open Field are of equal size. The trap was inserted into the dark tube, removed when the animal entered the tube and the external swing door was closed.

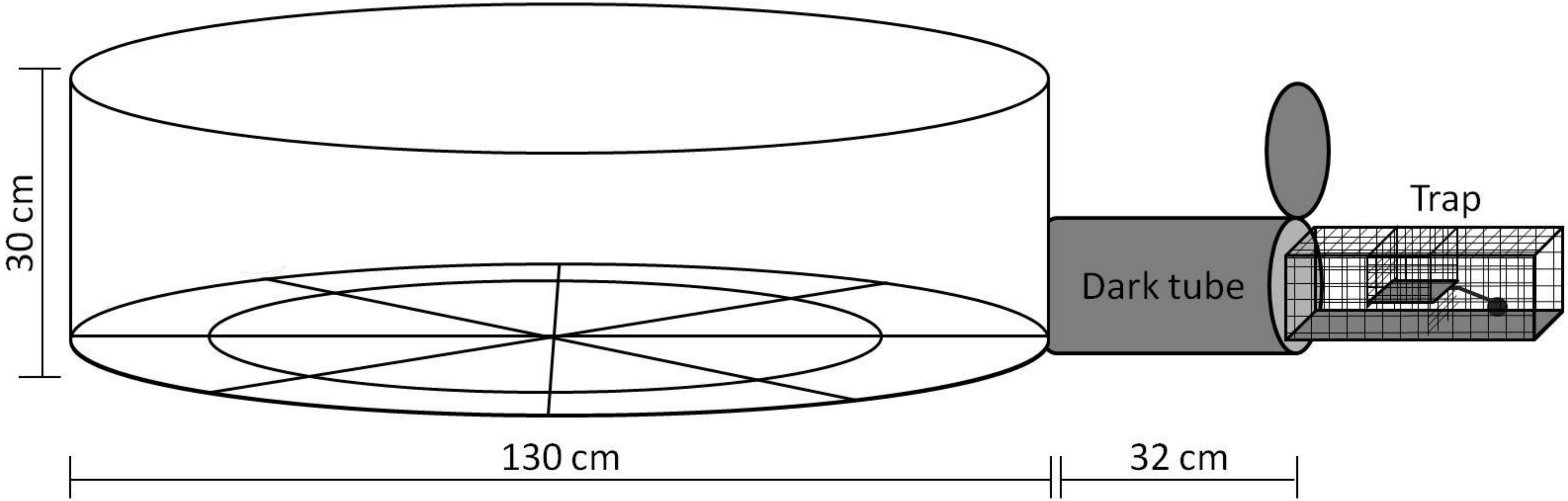

Fig. S2 – Repeatabilities of observed original behavioural variables for striped field mice (*Apodemus agrarius*) quantified in short behavioural tests on-site. Asterisks represent significance.

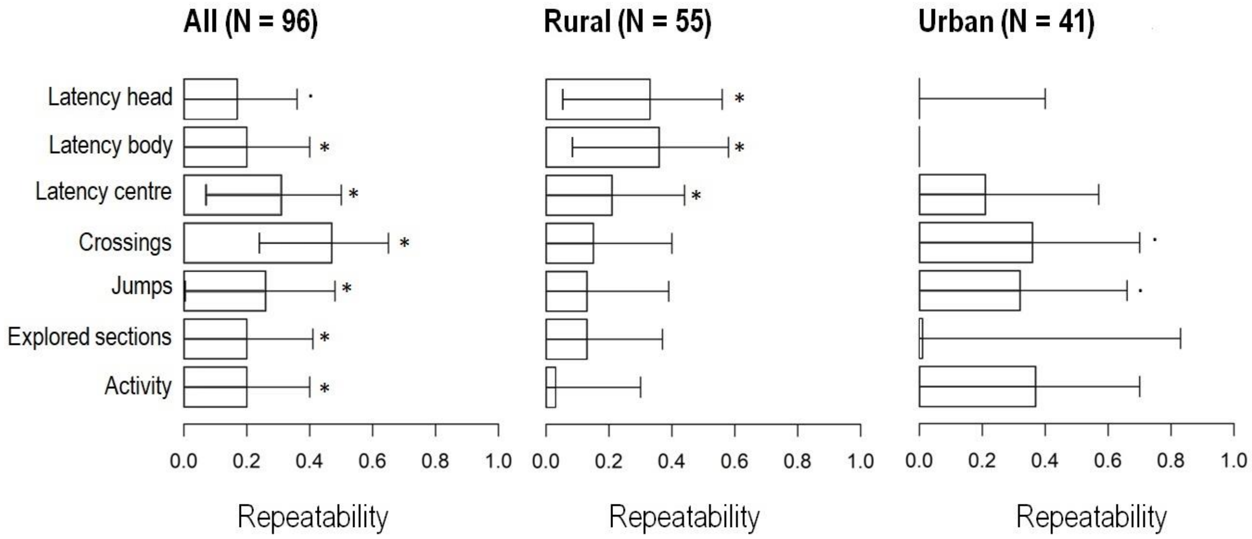

Table S1 – Latency to emerge from the tube with the head, latency to emerge from the tube with the whole body (without tail), latency to enter the central part of the arena, number of crossings, number of jumps and proportion of time spent active in relation to habitat type (rural vs urban), experimental day mean-centred for the individual (MCI) and mean-centred for the period (MCP) for 96 individual striped field mice (*Apodemus agrarius*). Results relative to the variable "Explored sections" are not reported because the model failed to converge. Statistically significant effects are highlighted in bold font.

Variable	Model type	Transformation	Fixed factors	Estimate	SE	t/Z	P
Latency head	Gaussian	log10	Intercept	2.88	0.19	14.91	< 0.001
			Habitat type (Urban)	-0.15	0.33	-0.46	0.65
			DayMCI	-0.006	0.02	-0.36	0.72
			DayMCP	0.01	0.01	1.48	0.14
Latency body	Gaussian	log10	Intercept	3.42	0.17	19.57	< 0.001
			Habitat type (Urban)	-0.14	0.30	-0.49	0.25
			DayMCI	0.003	0.02	0.21	0.39
			DayMCP	0.01	0.01	1.59	0.12
Latency centre	Gaussian	log	Intercept	3.83	0.13	30.05	< 0.001
			Habitat type (Urban)	-0.98	0.22	-4.48	**< 0.001**
			DayMCI	0.01	0.01	1.23	0.14
			DayMCP	0.002	0.004	0.44	0.66
Crossing	Poisson	/	Intercept	1.87	0.07	27.84	< 0.001
			Habitat type (Urban)	0.62	0.106	5.89	**< 0.001**
			DayMCI	0.01	0.005	1.35	0.18
			DayMCP	-0.004	0.002	-1.69	0.09
Jump	Poisson	/	Intercept	2.30	0.11	21.33	< 0.001
			Habitat type (Urban)	0.24	0.17	1.38	0.17
			DayMCI	-0.003	0.01	-0.51	0.61
			poly(DayMCP, 2)1	1.07	1.25	0.86	0.39
			poly(DayMCP, 2)2	1.66	0.65	2.56	**0.01**
General activity	Gaussian	asin-sqrt	Intercept	1.10	0.03	39.19	< 0.001
			Habitat type (Urban)	0.11	0.05	2.25	**0.02**
			DayMCI	-0.002	0.003	-0.75	0.44
			DayMCP	0.0001	0.001	0.06	0.95
Spatial exploration (PC1)	Gaussian	/	Intercept	-0.23	0.11	-2.19	0.03
			Habitat type (Urban)	0.73	0.18	4.07	**< 0.001**
			DayMCI	0.0003	0.01	0.03	0.97
			DayMCP	0.001	0.004	0.23	0.82
Boldness (PC2)	Gaussian	/	Intercept	-0.25	0.10	-2.37	0.02
			Habitat type (Urban)	0.55	0.18	3.10	**0.003**
			DayMCI	-0.01	0.01	-0.71	0.48
			DayMCP	-0.005	0.004	-1.32	0.19

Table S2 – Latency to emerge from the tube with the head, latency to emerge from the tube with the whole body (without tail), latency to enter the central part of the arena, number of crossings, number of jumps, proportion of explored sections and proportion of time spent active in relation to sealed surface in a 1km buffer, experimental day mean-centred for the individual (MCI) and mean-centred for the period (MCP) for 96 individual striped field mice (*Apodemus agrarius*). Statistically significant effects are highlighted in bold font.

Variable	Model type	Transformation	Fixed factors	Estimate	SE	t/Z	P
Latency head	Gaussian	log10	Intercept	1.31	0.08	334.53	< 0.001
			Sealing	-0.01	0.004	4.72	**0.03**
			DayMCI	-0.001	0.01	0.03	0.86
			DayMCP	0.003	0.003	0.99	0.32
Latency body	Gaussian	log 10	Intercept	3.52	0.18	590.41	< 0.001
			Sealing	-0.01	0.01	4.50	**0.04**
			DayMCI	0.006	0.02	0.74	0.39
			DayMCP	0.007	0.006	1.35	0.25
Latency centre	Gaussian	log	Intercept	3.86	0.13	1197.83	< 0.001
			Sealing	-0.03	0.01	26.19	**< 0.001**
			DayMCI	0.01	0.01	2.30	0.13
			DayMCP	0.001	0.004	0.05	0.83
Crossing	Poisson	/	Intercept	1.83	0.06	28.46	< 0.001
			Sealing	0.02	0.003	7.24	**< 0.001**
			DayMCI	0.005	0.005	1.01	0.31
			DayMCP	-0.002	0.002	-1.00	0.32
Jump	Poisson	/	Intercept	2.40	0.11	21.24	< 0.001
			Sealing	-0.0001	0.01	-0.02	0.99
			DayMCI	-0.001	0.01	-0.15	0.88
			poly(DayMCP, 2)1	0.42	1.29	0.32	0.75
			poly(DayMCP, 2)2	1.64	0.65	2.52	**0.01**
Explored sections	Binomial	/	Intercept	-0.67	0.242	-2.79	0.005
			Sealing	0.05	0.013	3.68	**< 0.001**
			DayMCI	-0.01	0.024	-0.35	0.72
			DayMCP	-0.01	0.008	-1.48	0.14
General activity	Gaussian	asin-sqrt	Intercept	1.11	0.03	2641.81	< 0.001
			Sealing	0.002	0.001	3.08	0.08
			DayMCI	-0.0018	0.003	0.60	0.44
			DayMCP	-0.0001	0.001	0.01	0.92
Spatial exploration (PC1)	Gaussian	/	Intercept	-0.19	0.11	-1.71	0.09
			Sealing	0.02	0.01	3.08	**0.003**
			DayMCI	0.001	0.01	0.10	0.92
			DayMCP	0.0003	0.004	0.07	0.95
Boldness (PC2)	Gaussian	/	Intercept	-0.32	0.10	-3.06	0.003
			Sealing	0.02	0.005	4.15	**< 0.001**
			DayMCI	-0.01	0.01	-0.91	0.37
			DayMCP	-0.003	0.003	-0.81	0.42

Table S3 – PCA loadings of behaviours in dark light and open field tests as well as repeatability for 96 striped field mice (*Apodemus agrarius*). Behaviours that contributed importantly to a component are highlighted in bold font.

Variable	PC 1	PC 2	Transformation for PCA
Latency body[a†]	0.13	**0.90**	Log10
Latency center[b†]	-0.29	**0.68**	Log
Crossing[c]	**0.56**	**-0.44**	/
Jump[d]	**0.93**	0.16	/
General activity[e]	**0.70**	-0.25	Sqrt, arcsine
Eigenvalue	1.75	1.57	
Variance explained (%)	35.1	31.4	
Repeatability (±SE)*	0.39 ± 0.1	0.26 ± 0.11	
*P**	< 0.001	0.013	

[a]Latency to leave the dark tube with the entire body (without tail) and enter the open field arena.
[b]Latency to enter the central exposed area of the open field arena.
[c]Number of crossings into the central part of the open field arena
[d]Number of jumps.
[e]Proportion of time spent active in the open field arena.
†PCA scores resulting from these components were transformed inverting the signs, so that a higher score expresses higher boldness and a lower score expresses lower boldness.
*Repeatabilities and their significance were calculated after Lessells & Boag (1987) and Nakagawa & Schielzeth (2010) from a one-way ANOVA with individual as a factor using the R package rptR (Stoffel et al., 2017).

Table S4 – Repeatabilities of composite behavioural variables and observed original behavioural variables at dataset level and for urban and rural populations for 96 striped field mice (*Apodemus agrarius*).

Variable	Model type	All				Rural				Urban			
		R	SE	CI	P	R	SE	CI	P	R	SE	CI	P
Latency head	Gaussian	0.17	0.11	[0 - 0.36]	0.065	0.33	0.13	[0.05 - 0.56]	0.005	0	0.12	[0 - 0.40]	1
Latency body	Gaussian	0.20	0.10	[0 - 0.39]	0.033	0.36	0.13	[0.08 - 0.58]	0.003	0	0.0	[0 - 0]	0.5
Latency centre	Gaussian	0.31	0.11	[0.07 - 0.50]	0.003	0.21	0.12	[0 - 0.44]	0.050	0.21	0.17	[0 - 0.57]	0.178
Crossings	Poisson	0.47	0.11	[0.24 - 0.65]	< 0.001	0.15	0.12	[0 - 0.40]	0.147	0.36	0.19	[0 - 0.69]	0.061
Jumps	Poisson	0.26	0.12	[0.004 - 0.48]	0.011	0.13	0.12	[0 - 0.39]	0.158	0.32	0.20	[0 - 0.66]	0.085
Sections	Binary	0.20	0.11	[0 - 0.40]	0.039	0.13	0.11	[0 - 0.37]	0.158	0.01	0.18	[0 - 0.83]	0.467
General activity	Gaussian	0.20	0.11	[0 - 0.39]	0.046	0.03	0.09	[0 - 0.30]	0.442	0.37	0.2	[0 - 0.70]	0.050
Spatial exploration	Gaussian	0.39	0.10	[0.16 - 0.56]	< 0.001	0.25	0.12	[0 - 0.49]	0.024	0.27	0.2	[0 - 0.62]	0.103
Boldness	Gaussian	0.26	0.11	[0.02 - 0.45]	0.013	0.29	0.12	[0.04 - 0.51]	0.013	0	0.1	[0 - 0.42]	0.5

Table S5 – Mean, standard deviation and results of the Mann-Whitney-U test comparing the intercepts and slopes of 56 rural and urban striped field mice (*Apodemus agrarius*). Statistically significant effects are highlighted in bold font.

	Variable	Rural		Urban		W	P
		**Mean**	**SD**	**Mean**	**SD**		
Slopes	Latency head	-0.001	0.01	0.001	0.01	330	0.617
	Latency body	-0.002	0.02	0.004	0.01	287	0.217
	Latency centre	-0.005	0.02	0.01	0.02	196	**0.005**
	Crossings	-0.001	0.01	0.002	0.01	277	0.160
	Jumps	-0.003	0.02	0.01	0.01	258	0.083
	Sections	0.47	1.80	-0.83	1.17	511	**0.009**
	General activity	0.001	0.01	-0.002	0.01	438	0.187
	Spatial exploration	0.003	0.01	-0.01	0.01	490	**0.026**
	Boldness	0.004	0.02	-0.01	0.02	482	**0.037**
Intercepts	Latency head	0.005	0.14	-0.008	0.11	387	0.653
	Latency body	0.014	0.21	-0.023	0.16	416	0.346
	Latency centre	0.177	0.42	-0.31	0.53	554	**0.001**
	Crossings	-0.192	0.30	0.343	0.34	94	**< 0.001**
	Jumps	-0.088	0.31	0.16	0.33	212	**0.011**
	Sections	-21.38	59.75	38.55	48.05	164	**0.001**
	General activity	-0.018	0.08	0.032	0.09	212	**0.011**
	Spatial exploration	-0.196	0.38	0.35	0.56	154	**< 0.001**
	Boldness	-0.083	0.30	0.148	0.29	208	**0.009**

Table S6 – Latency to emerge from the tube with the head, latency to emerge from the tube with the whole body (without tail), latency to enter the central part of the arena, number of crossings, number of jumps, proportion of explored sections, proportion of time spent active, PC spatial exploration and boldness in relation to sealed surface in a 1km buffer, experimental day mean-centred for the individual (MCI) for 41 individual striped field mice (*Apodemus agrarius*). Results are relative to urban individuals only. Statistically significant effects are highlighted in bold font.

Variable	Model type	Transformation	Fixed factors	Estimate	SE	t/Z	P
Latency head	Gaussian	log10	Intercept	2.10	0.34	6.18	< 0.001
			**Sealing (1km)**	-0.03	0.009	-3.08	**0.002**
			DayMCI	0.034	0.03	1.00	0.316
Latency body	Gaussian	log	Intercept	4.86	0.68	7.19	< 0.001
			**Sealing (1km)**	-0.05	0.02	-2.75	**0.006**
			DayMCI	0.136	0.07	2.01	0.045
Latency centre	Gaussian	log	Intercept	3.43	0.61	5.60	< 0.001
			**Sealing (1km)**	-0.02	0.02	-1.06	0.289
			DayMCI	0.06	0.05	1.07	0.284
Crossing	Gaussian	/	Intercept	4.73	2.84	1.67	0.096
			**Sealing (1km)**	0.28	0.078	3.52	**< 0.001**
			DayMCI	0.027	0.245	0.11	0.912
Jump	Poisson	/	Intercept	3.50	0.42	8.42	< 0.001
			**Sealing (1km)**	-0.0297	0.01	-2.55	**0.011**
			DayMCI	-0.006	0.01	-0.58	0.565
Explored sections	Binomial	/	Intercept	-0.50	0.901	-0.56	0.579
			Sealing (1km)	0.05	0.026	1.89	0.059
			DayMCI	-0.06	0.109	-0.54	0.592
General activity	Gaussian	asin-sqrt	Intercept	1.35	0.13	10.66	< 0.001
			Sealing (1km)	-0.004	0.003	-1.13	0.260
			DayMCI	-0.0169	0.009	-1.89	0.058
Spatial exploration	Gaussian	/	Intercept	1.03	0.52	1.98	0.048
			Sealing (1km)	-0.02	0.01	-1.10	0.269
			DayMCI	-0.014	0.04	-0.33	0.741
Boldness	Gaussian	/	Intercept	-0.83	0.44	-1.90	0.057
			**Sealing (1km)**	0.04	0.012	2.94	**0.003**
			DayMCI	-0.07	0.04	-1.68	0.092

Table S7 – Mean, standard deviation and results of the Mann-Whitney-U test comparing the behavioural responses of the 42 rural and 25 urban striped field mice (*Apodemus agrarius*) tested once in 2018, and of all the three sampling years pooled together. This is the only case in which data from 2018 were used or added and are only meant to help disentangle year and area effects. Statistically significant effects are highlighted in bold font.

	Variable	Rural		Urban		W	P
		Mean	SD	Mean	SD		
2018 only	Latency head	45.52	57.19	40.88	33.73	476	0.529
	**Latency body**	130.98	95.99	59.60	39.13	757.5	**0.003**
	**Latency centre**	121.98	82.36	61.12	79.77	808.5	**< 0.001**
	**Crossings**	1.64	1.34	5.36	2.46	77.5	**< 0.001**
	Jumps	3.88	3.42	4.72	4.25	480	0.562
	**Sections**	0.80	0.20	0.93	0.12	288	**0.001**
	**General activity**	0.67	0.22	0.79	0.19	350.5	**0.024**
	**Spatial exploration**	-0.17	0.88	0.28	1.13	388	0.077
	**Boldness**	-0.48	0.76	0.81	0.82	133	**< 0.001**
All years pooled together	Latency head	52.34	72.99	51.62	80.14	6778	0.499
	**Latency body**	88.44	91.79	63.31	81.29	7565.5	**0.025**
	**Latency centre**	89.95	84.31	43.39	60.01	9350	**< 0.001**
	**Crossings**	5.40	4.35	11.75	7.11	2955	**< 0.001**
	**Jumps**	9.08	8.72	13.28	13.29	5378.5	**0.035**
	**Sections**	0.84	0.19	0.95	0.10	3788	**< 0.001**
	**General activity**	0.73	0.21	0.82	0.19	4574	**< 0.001**
	**Spatial exploration**	-0.25	0.83	0.43	1.11	3901	**< 0.001**
	**Boldness**	-0.30	0.86	0.52	1.01	3481.5	**< 0.001**

Table S8 – Repeatabilities of composite behavioural variables and observed original behavioural variables at dataset level and for urban and rural populations for 96 striped field mice (*Apodemus agrarius*) for the first two repeats of the behavioural test.

Variable	Model type	All (round 1-2- only)				Rural (round 1-2- only)				Urban (round 1-2- only)			
		R	SE	CI	P	R	SE	CI	P	R	SE	CI	P
Latency head	Gaussian	0.18	0.12	[0 - 0.42]	0.094	0.48	0.13	[0.18 - 0.68]	0.002	0	0.13	[0 - 0.45]	1
Latency body	Gaussian	0.25	0.12	[0 - 0.48]	0.038	0.51	0.12	[0.25 - 0.71]	0.001	0	0.1	[0 - 0.46]	1.0
Latency centre	Gaussian	0.26	0.12	[0.01 - 0.49]	0.018	0.19	0.14	[0 - 0.48]	0.119	0.16	0.17	[0 - 0.54]	0.228
Crossings	Poisson	0.49	0.11	[0.26 - 0.68]	< 0.001	0.13	0.13	[0 - 0.43]	0.240	0.43	0.19	[0 - 0.72]	0.029
Jumps	Poisson	0.19	0.12	[0 - 0.43]	0.081	0.03	0.10	[0 - 0.34]	0.441	0.36	0.20	[0 - 0.71]	0.066
Sections	Binary	0.14	0.08	[0 - 0.28]	0.093	0.10	0.09	[0 - 0.31]	0.223	0.08	0.31	[0 - 0.98]	0.321
General activity	Gaussian	0.18	0.12	[0 - 0.41]	0.087	0.04	0.11	[0 - 0.35]	0.424	0.35	0.2	[0 - 0.67]	0.053
Spatial exploration	Gaussian	0.34	0.12	[0.09,0.55]	0.003	0.16	0.13	[0 - 0.45]	0.192	0.30	0.2	[0 - 0.64]	0.074
Boldness	Gaussian	0.26	0.12	[0.01 - 0.48]	0.035	0.37	0.14	[0.05 - 0.62]	0.011	0	0.1	[0 - 0.43]	1.0

Table S9 – Latency to emerge from the tube with the head, latency to emerge from the tube with the whole body (without tail), latency to enter the central part of the arena, number of crossings, number of jumps, proportion of time spent active, PC spatial exploration and boldness in relation to habitat type (rural vs urban), experimental day mean-centred for the individual (MCI) and mean-centred for the period (MCP) for 96 individual striped field mice (*Apodemus agrarius*). Results are relative to the first two tests only. Statistically significant effects are highlighted in bold font.

Variable	Model type	Transformation	Fixed factors	Estimate	SE	t/Z	P
Latency head	Gaussian	log10	Intercept	2.79	0.21	13.44	< 0.001
			Habitat type (Urban)	-0.05	0.34	-0.14	0.890
			DayMCI	-0.017	0.02	-0.84	0.407
			DayMCP	0.01	0.01	1.52	0.134
Latency body	Gaussian	log10	Intercept	3.34	0.19	17.63	< 0.001
			Habitat type (Urban)	-0.06	0.31	-0.20	0.843
			DayMCI	-0.005	0.02	-0.29	0.770
			DayMCP	0.01	0.01	1.64	0.107
Latency centre	Gaussian	log	Intercept	3.81	0.13	28.34	< 0.001
			**Habitat type (Urban)**	-0.95	0.22	-4.28	**< 0.001**
			DayMCI	0.01	0.01	0.90	0.374
			DayMCP	0.002	0.005	0.49	0.624
Crossing	Poisson	/	Intercept	1.88	0.07	26.98	< 0.001
			**Habitat type (Urban)**	0.60	0.107	5.63	**< 0.001**
			DayMCI	0.01	0.005	1.26	0.210
			DayMCP	-0.004	0.002	-1.73	0.084
Jump	Poisson	/	Intercept	2.31	0.11	21.21	< 0.001
			Habitat type (Urban)	0.23	0.17	1.30	0.194
			DayMCI	0.000	0.01	0.00	0.996
			poly(DayMCP,2)1	0.93	1.09	0.85	0.395
			**poly(DayMCP,2)2**	1.85	0.66	2.79	**0.005**
General activity	Gaussian	asin-sqrt	Intercept	1.11	0.03	36.62	< 0.001
			**Habitat type (Urban)**	0.10	0.05	2.02	**0.046**
			DayMCI	-0.001	0.003	-0.32	0.748
			DayMCP	0.0003	0.001	0.25	0.806
Spatial exploration	Gaussian	/	Intercept	-0.18	0.11	-1.68	0.096
			**Habitat type (Urban)**	0.69	0.18	3.80	**< 0.001**
			DayMCI	0.0077	0.01	0.73	0.467
			DayMCP	0.002	0.004	0.43	0.668
Boldness	Gaussian	/	Intercept	-0.22	0.11	-1.98	0.050
			**Habitat type (Urban)**	0.52	0.19	2.78	**0.007**
			DayMCI	0.00	0.01	-0.39	0.698
			DayMCP	-0.005	0.004	-1.39	0.171

Table S10 – Latency to emerge from the tube with the head, latency to emerge from the tube with the whole body (without tail), latency to enter the central part of the arena, number of crossings, number of jumps, proportion of explored sections, proportion of time spent active, PC spatial exploration and boldness in relation to sealed surface in a 1 km buffer, experimental day mean-centred for the individual (MCI) and mean-centred for the period (MCP) for 96 individual striped field mice (*Apodemus agrarius*). Results are relative to the first two tests only. Statistically significant effects are highlighted in bold font.

Variable	Model type	Transformation	Fixed factors	Estimate	SE	t/Z	P
Latency head	Gaussian	log10	Intercept	1.27	0.09	13.93	< 0.001
			Sealing (1km)	-0.01	0.004	-1.31	0.194
			DayMCI	-0.006	0.01	-0.63	0.533
			DayMCP	0.003	0.003	1.02	0.314
Latency body	Gaussian	log10	Intercept	1.49	0.08	17.89	< 0.001
			Sealing (1km)	-0.01	0.00	-1.85	0.067
			DayMCI	0.002	0.01	0.22	0.829
			DayMCP	0.008	0.006	1.20	0.236
Latency centre	Gaussian	log	Intercept	3.84	0.14	28.03	< 0.001
			**Sealing (1km)**	-0.03	0.01	-4.42	**< 0.001**
			DayMCI	0.01	0.01	0.94	0.351
			DayMCP	0.001	0.005	0.23	0.822
Crossing	Poisson	/	Intercept	1.84	0.07	27.54	< 0.001
			**Sealing (1km)**	0.02	0.003	6.91	**< 0.001**
			DayMCI	0.005	0.005	1.01	0.311
			DayMCP	-0.002	0.002	-1.03	0.304
Jump	Poisson	/	Intercept	2.87	0.38	7.61	< 0.001
			Sealing (1km)	-0.0153	0.02	-0.82	0.412
			DayMCI	0.018	0.01	1.38	0.168
			poly(DayMCP, 2)1	-5.30	4.00	-1.32	0.186
			**poly(DayMCP, 2)2**	2.54	0.79	3.21	**0.001**
Explored sections	Binomial	/	Intercept	-0.57	0.265	-2.15	0.031
			**Sealing (1km)**	0.05	0.014	3.23	**0.001**
			DayMCI	0.00	0.027	0.14	0.890
			DayMCP	-0.01	0.009	-1.46	0.145
General activity	Gaussian	asin-sqrt	Intercept	1.12	0.03	35.86	< 0.001
			Sealing (1km)	0.002	0.001	1.38	0.170
			DayMCI	-0.0007	0.003	-0.23	0.822
			DayMCP	0.0001	0.001	0.08	0.934
Spatial exploration	Gaussian	/	Intercept	-0.14	0.11	-1.25	0.213
			**Sealing (1km)**	0.02	0.01	2.87	**0.005**
			DayMCI	0.009	0.01	0.82	0.414
			DayMCP	0.0010	0.004	0.26	0.796
Boldness	Gaussian	/	Intercept	-0.29	0.11	-2.65	0.010
			**Sealing (1km)**	0.02	0.005	3.84	**< 0.001**
			DayMCI	-0.01	0.01	-0.59	0.560
			DayMCP	-0.003	0.004	-0.86	0.394

Table S11 – PC spatial exploration and single behavioural variables referring to explorative behaviour (number of crossings, number of jumps, proportion of explored sections and proportion of time spent active in relation to habitat type (rural vs urban), experimental day mean-centred for the period (MCP) for 96 individual striped field mice (*Apodemus agrarius*). Results are relative to the first test only. Statistically significant effects are highlighted in bold font.

Variable	Model type	Transformation	Fixed factors	Estimate	SE	t/Z	P
Crossing	Poisson	/	Intercept	1.93	0.05	35.59	< 0.001
			**Habitat type (Urban)**	0.67	0.070	9.62	**< 0.001**
			DayMCP	-0.002	0.002	-1.06	0.288
Jump	Poisson	/	Intercept	2.63	0.04	69.07	< 0.001
			**Habitat type (Urban)**	0.3065	0.06	5.31	**< 0.001**
			DayMCP	0.006	0.00	4.10	< 0.001
Explored sections	Gaussian	asin-sqrt	Intercept	-0.42	0.308	-1.36	0.174
			**Habitat type (Urban)**	1.17	0.487	2.40	**0.016**
			DayMCP	-0.02	0.011	-2.00	0.046
General activity	Gaussian	asin-sqrt	Intercept	1.18	0.04	31.35	< 0.001
			Habitat type (Urban)	0.099	0.057	1.72	0.089
			DayMCP	0.0017	0.001	1.22	0.224
Spatial exploration	Gaussian	/	Intercept	-0.05	0.14	-0.35	0.728
			**Habitat type (Urban)**	0.67	0.22	3.09	**0.003**
			DayMCP	0.006	0.01	1.12	0.267

Table S12 – PC spatial exploration and single behavioural variables referring to explorative behaviour (number of crossings, number of jumps, proportion of explored sections and proportion of time spent active in relation to sealed surface in a 1km buffer and experimental day mean-centred for the period (MCP) for 96 individual striped field mice (*Apodemus agrarius*). Results are relative to the first test only. Statistically significant effects are highlighted in bold font.

Variable	Model type	Transformation	Fixed factors	Estimate	SE	t/Z	P
Crossing	Poisson	/	Intercept	1.92	0.05	36.34	< 0.001
			**Sealing (1km)**	0.021	0.00	10.45	**< 0.001**
			DayMCP	0.001	0.00	0.50	0.615
Jump	Poisson	/	Intercept	2.68	0.04	70.58	< 0.001
			Sealing (1km)	0.00	0.00	0.20	0.844
			poly(DayMCP, 2)1	0.66	0.29	2.29	0.022
			poly(DayMCP, 2)2	-0.54	0.28	-1.97	0.049
Explored sections	Binomial	/	Intercept	-0.434	0.30	-1.430	0.153
			**Sealing (1km)**	0.036	0.01	2.43	**0.015**
			DayMCP	-0.021	0.01	-1.79	0.073
General activity	Gaussian	asin-sqrt	Intercept	1.19	0.04	31.21	< 0.001
			Sealing (1km)	0.002	0.00	1.29	0.199
			DayMCP	0.002	0.00	1.14	0.257
Spatial exploration	Gaussian	/	Intercept	0.01	0.15	0.04	0.969
			**Sealing (1km)**	0.01	0.01	2.159	**0.033**
			DayMCP	0.005	0.01	0.94	0.351
